# Psychometric properties of the transaddiction craving triggers questionnaire in alcohol use disorder

**DOI:** 10.1002/mpr.1815

**Published:** 2019-12-29

**Authors:** Cora von Hammerstein, Aurélien Cornil, Stéphane Rothen, Lucia Romo, Yasser Khazaal, Amine Benyamina, Joël Billieux, Amandine Luquiens

**Affiliations:** ^1^ Department of Psychiatry and Addictology, University Paris‐Saclay, University Paris‐Sud, UVSQ, CESP, INSERM U 1178, APHP Paul Brousse Hospital Villejuif France; ^2^ EA 4430 CLIPSYD University Paris Nanterre Nanterre France; ^3^ Laboratory for Experimental Psychopathology (LEP), Psychological Science Research Institute Université catholique de Louvain Louvain‐la‐Neuve Belgium; ^4^ Research Center for Statistics, Geneva School of Economics and Management University of Geneva Geneva Switzerland; ^5^ Inserm, U894 Center for Psychiatry and Neuroscience Paris France; ^6^ Addiction Medecine, Department of psychiatry Lausanne University Hospitals Lausanne Switzerland; ^7^ Addictive and Compulsive Behaviours Lab. Institute for Health and Behaviour University of Luxembourg Esch‐sur‐Alzette Luxembourg; ^8^ Institute of Psychology University of Lausanne Lausanne Switzerland; ^9^ Universitary Hospital of Nîmes Paris France

**Keywords:** alcohol use disorder, craving, psychometrics, triggers, validation

## Abstract

**Objectives:**

We aimed to develop the transaddiction craving triggers questionnaire (TCTQ), which assesses the propensity of specific situations and contexts to trigger craving and to test its psychometric properties in alcohol use disorder (AUD).

**Methods:**

This study included a sample of 111 AUD outpatients. We performed exploratory factor analysis (EFA) and calculated item–dimension correlations. Internal consistency was measured with Cronbach's alpha coefficient. Construct validity was assessed through Spearman correlations with craving, emotional symptoms, impulsivity, mindfulness, and drinking characteristics.

**Results:**

The EFA suggested a 3‐factor solution: unpleasant affect, pleasant affect, and cues and related thoughts. Cronbach's coefficient alpha ranged from .80 to .95 for the three factors and the total score. Weak positive correlations were identified between the TCTQ and drinking outcomes, and moderate correlation were found between the TCTQ and craving strength, impulsivity, anxiety, depression, and impact of alcohol on quality of life.

**Conclusions:**

The 3‐factor structure is congruent with the well‐established propensity of emotions and cues to trigger craving. Construct validity is supported by close relations between the TCTQ and psychological well‐being rather than between the TCTQ and drinking behaviors. Longitudinal validation is warranted to assess sensitivity to change of the TCTQ and to explore its psychometric properties in other addictive disorders.

## 1 INTRODUCTION

In the 5th edition of the *Diagnostic and Statistical Manual of Mental Disorders* (*DSM‐5*; American Psychiatric Association, 2013), craving was added as one of 12 criteria used to define alcohol use disorder (AUD). Previous studies have consistently shown that craving is positively correlated with AUD severity, drinking outcomes, and related negative consequences (Chakravorty et al., 2010; Murphy, Stojek, Few, Rothbaum, & Mackillop, 2014). Furthermore, craving is known as a robust predictor of relapse within different contexts in substance use disorders (Kavanagh et al., 2013; Oslin, Cary, Slaymaker, Colleran, & Blow, 2009). Initial definitions of craving referred to a motivational state characterized by an intense “urge to consume” a psychoactive substance (Baker, Morse, & Sherman, 1986). Some authors suggested that the term *craving* refers to the desire of experiencing the effects of a drug, whereas *urge* refers to the behavioral intention to use a drug (Marlatt & Gordon, 1985, p. 200; Sayette et al., 2000). Other models hold that craving and urge belong to a continuum of desire in which craving is located at the extreme pole (Kavanagh, Andrade, & May, 2005). Most researchers have defined craving as a subjective motivational state related to the desire to use a drug (Kassel & Shiffman, 1992). Tiffany and Drobes (1991) proposed a broader definition of craving, giving consideration to the behavioral intention to use the substance and the anticipation of its positive and negative reinforcing effects, in line with the theory of social learning (Bandura, 1978, 1985; Bandura, Ross, & Ross, 1963). More recently, Kavanagh and colleagues formulated a cognitive model of craving: the elaborated intrusion theory of desire (EIT; Kavanagh et al., 2005). According to this theory, craving is a process, common to all addictions, in which the desire for a specific target (a substance or a behavior) and its expected effects overwhelm the attentional capacities of an individual. According to this theory, craving is the consequence of cognitive elaborations (involving mental imageries and verbal thoughts) triggered by specific environmental contexts, physiological sensations, negative and positive emotions, or associated thoughts.

The lack of consensual conceptualization and definition of craving complicates its measurement. Single‐item assessment of craving with a Likert scale or visual analog scales has proved easy to implement but insufficient to capture the various conceptualizations of craving (Sayette et al., 2000). Several multi‐item tools have thus been developed in an effort to assess the different components of craving beyond frequency and intensity (Flannery, Volpicelli, & Pettinati, 1999). For example, various instruments were created to target the intrusive features of craving (May et al., 2014), its compulsive and obsessive components (Anton, Moak, & Latham, 1995), or the various dimensions related to desire and intension to drink, along with the expectations related to positive and negative reinforcement (Bohn, Krahn, & Staehler, 1995). Measurement of the duration of a craving episode is still a matter of debate (Heishman, Lee, Taylor, & Singleton, 2010; Heishman, Saha, & Singleton, 2004; Heishman, Singleton, & Moolchan, 2003), as is the recall period to allow for a reliable measure (Shiffman, 2000a). In particular, it has been suggested that assessing craving over a specific time frame might not be representative, given that craving is highly fluctuant and context dependent (Childress et al., 1993; Conklin & Tiffany, 2002).

Various internal cues (e.g., affective states and physical sensations) and external cues (e.g., environmental or contextual factors) are known to be capable of triggering craving episodes (Carter & Tiffany, 1999; George et al., 2001; Kavanagh et al., 2005; Thomas, Drobes, & Deas, 2005; Witteman et al., 2015). For example, it is well documented that external cues (e.g., a bottle of wine, an advertisement, or a bar) are efficient at triggering craving (George et al., 2001; Kavanagh et al., 2005; Schacht, Anton, & Myrick, 2013; Thomas et al., 2005; Witteman et al., 2015). The same applies to internal cues, such as negative mood, which have consistently been described as craving triggers (Cooney, Litt, Morse, Bauer, & Gaupp, 1997; Ehlers, Gilder, Gizer, & Wilhelmsen, 2018; Wheeler et al., 2008). More generally, emotion regulation can play a pivotal role in the onset, perpetuation, and relapse of AUD (Volkow, Wang, Fowler, & Tomasi, 2012). From this perspective, drinking can be viewed as a maladaptive regulation strategy that aims to regulate both negative and positive emotions (Cooper, Frone, Russell, & Mudar, 1995; Shafiei, Hoseini, Bibak, & Azmal, 2014). According to the EIT (Kavanagh et al., 2005), different types of craving triggers can be identified, including negative affect, physiological deficits, external cues, related thoughts that can reach the object of addiction by ricochet, and anticipatory responses (e.g., salivation). The EIT posits that the above‐mentioned triggers can induce a craving episode through the elaboration of “desire thoughts” consisting of mental imageries (e.g., mentally picturing a drinking episode) and verbal thoughts (e.g., “How nice it would be to drink alcohol right now!”). According to the EIT, a double vicious circle is involved in the craving experience. The first vicious circle is directly related to the pleasure (positive reinforcement) and relief (negative reinforcement) provoked by the mental imagery process per se. The second vicious circle implies a counterfactual process (i.e., a comparison between the desired and the actual state) that promotes a sense of deficit (e.g., alcohol withdrawal) and reinforces the vividness of the craving experience.

Given the robust association between craving and relapse, most evidence‐based psychological interventions tend to focus on the identification of high‐risk situations, namely, situations that are supposed to trigger craving, such as unpleasant or pleasant emotions, social pressure, urges and temptations, tests of personal control, or conflicts with others (Marlatt & Gordon, 1985; Shafiei et al., 2014). A crucial aspect of psychological intervention is thus to help individuals develop the ability to avoid triggers or to learn skills or adaptive strategies to efficiently cope with them (Bowen, Chawla, & Marlatt, 2011; Marlatt & Gordon, 1985).

Beyond the measurement of various features of craving and its intensity or frequency, it therefore appears essential to develop instruments that can measure craving in clinical settings (e.g., to identify a patient's relevant triggers and high‐risk situations and to assess the effect of an intervention that targets craving). As no comparable instrument is to our knowledge available to date in any addiction, the current study thus aimed to develop a new self‐report that assesses craving triggers whatever the addictive behavior/substance, the transaddiction craving triggers questionnaire (TCTQ), to test its psychometric properties in a sample of AUD outpatients, and to investigate its construct validity. Craving trigger could be associated with psychological discomfort and with restriction of activities in an effort to avoid them when self‐efficacy is low. Craving trigger could thus be associated with the impact of alcohol on quality of life. Moreover, resisting craving and being less sensitive to craving triggers could involve protective psychological factors, such as high self‐control (reflecting less impulsive behaviors) or high trait mindfulness. Several previous studies have shown negative associations between trait mindfulness and craving for psychoactive drugs (Garland, Roberts‐Lewis, Kelley, Tronnier, & Hanley, 2014; Szeto, Schoenmakers, van de Mheen, Snelleman, & Waters, 2019; Tapper, 2018). As mindfulness is typically defined as a nonreactive, nonjudgmental form of metacognitive attention to the present moment, it is likely that the attention given to the present moment and the observation of one's own cognitions has an impact on the automated relationship between the trigger and the onset of craving. According to the EIT, cues associated with physiological responses lead to intrusive thoughts. When these intrusive thoughts result in intense emotional responses or a sense of deficit (e.g., alcohol withdrawal), it is likely that the related cognitive elaboration and mental imagery directly translate into a craving episode. Accordingly, being able to detach ourselves from our own automatic cognitive processes, a core feature of mindfulness is likely to reduce the duration of the craving and mitigate its intensity. The “present‐moment attention” that characterizes mindfulness and the metacognitive ability to see one's thoughts as simple thoughts and not as reflecting reality has been suggested to help interrupt the elaboration process of craving (Tapper, 2018). Furthermore, previous studies have shown that trait mindfulness is a strong predictor for attentional bias toward alcohol cues. Indeed, a high mindfulness trait was found to negatively correlate with attentional bias toward alcohol cues, and alcohol‐dependent patients with high trait mindfulness levels display less attentional bias and craving than do patients with low trait mindfulness (Garland, Boettiger, Gaylord, Chanon, & Howard, 2012). As attentional biases have been shown to increase craving, and the increase in craving can cause attentional biases (Field et al., 2016; Field & Cox, 2008), the positive impact of mindfulness on attentional biases is likely to reduce the appearance of craving following confrontation with triggers. Thus, according to the existing corpus of data that links mindfulness to craving, individual differences in the mindfulness trait constitute a theoretically sound correlate to establish the construct validity of the TCTQ. Indeed, the relationship of craving triggers and these concepts has not been investigated per se in the absence of an appropriate instrument to measure craving triggers.

## 2 MATERIALS AND METHODS

### 2.1 Population

Participants were recruited in the addiction facility of the Paul Brousse Hospital of Villejuif (France). All outpatients with current or remitted AUD (clinically diagnosed) attending care in a specialized addiction department were considered eligible for the study. No exclusion criterion was applied. Patients were informed and gave consent that the assessments conducted and the information included in the medical record could be used for research purposes. A total of 111 outpatients currently being treated for an AUD, comprising currently abstinent patients and patients controlling their consumption, were included in the study.

### 2.2 Ethics

Because interventions and assessments of the initial study were part of the patient's standard treatment, this observational noninterventional study met the French requirements of reference methodology M‐003, authorizing observational studies on medical data. All patients of the facility were systematically informed that their medical data could be used for research purposes. Patients were informed in writing that data from their medical record would be used for the present study. This study was approved by the French National Committee for Informatics and Liberty under number 2200863 v 0.

### 2.3 Measures

Sociodemographic characteristics were collected, including age, gender, and employment status. Patients completed all questionnaires in a single session. The instruction given before the completion of the questionnaires was to use the past month as a recall period.

The TCTQ is based on the EIT (Kavanagh et al., 2005), a model that has been shown to be relevant to account for substance and gambling cravings (Cornil et al., 2018; May, Kavanagh, & Andrade, 2015). The items of the TCTQ were generated on the basis of previous studies anchored in the EIT (May, Andrade, Panabokke, & Kavanagh, 2004) and an in‐depth qualitative study that applied the EIT to explore the phenomenology of gambling craving (Cornil et al., 2018). An initial list of 43 (see Supporting Information) was generated by one of the authors (A.C.) and validated by another author (J.B.). Each item was scored on a 6‐point Likert scale ranging from 1 (*not at all*) to 6 (*absolutely*). The items were divided into seven a priori categories inspired by the EIT: external cues (8 items), anticipatory responses (5 items), associated thoughts (5 items), physiological deficit (5 items), negative affect (9 items), positive affect (8 items), and sense of associated deficit (3 items). Unlike the Inventory of Drinking Situations (Annis, Graham, & Davis, 1982) and its short version (Isenhart, 1993), the TCTQ focuses on the internal and external cues that might be related to a drinking situation, as well as to other situations. These cues are proximal triggers of craving, which can directly lead to consumption.

Patients included in the study completed the TCTQ in the health care setting without any assistance.

In addition, patients completed the alcohol timeline followback (Sobell & Sobell, 1992) to assess drinking outcomes, the alcohol quality of life scale (Luquiens et al., 2016) to assess the impact of alcohol on quality of life, the Craving Experience Questionnaire (May et al., 2014) to assess craving frequency (CEQ‐F) and strength (CEQ‐S), the Five Facets Mindfulness Questionnaire (FFMQ; Heeren, Douilliez, Peschard, Debrauwere, & Philippot, 2011) to assess mindfulness levels, the short version of the urgency, premeditation, perseverance, sensation seeking, and positive urgency impulsive behavior scale (Billieux et al., 2012) to assess impulsivity, the Beck Depression Inventory (Bourque & Beaudette, 1982) to assess depression, and the Beck Anxiety Inventory (Freeston, Ladouceur, Thibodeau, Gagnon, & Rhéaume, 1994) to assess anxiety (see Table 1)

**Table 1:** Questionnaire description

Scale	Description	Internal consistency Cronbach's alpha	Authors
CEQ‐S	Strength of the last craving (10 items)	0.91	May et al. (2014)
*CEQ‐S intensity*	Intensity of craving	NA	
*CEQ‐S imagery*	Vividness of desire‐related imagery	NA	
*CEQ‐S intrusion*	Salience or dismissability of related intrusive thought	NA	
CEQ‐F	Craving frequency over the last weeks (10 items)	0.94	
*CEQ‐F intensity*	Intensity of craving	NA	
*CEQ‐F imagery*	Vividness of desire‐related imagery	NA	
*CEQ‐F intrusion*	Salience or dismissability of related intrusive thoughts	NA	
BAI	Anxiety severity, including physical symptoms (21 items)	.85*	Freeston et al. (1994)
BDI‐21	Depression severity (21 items)	.92^a^	Bourque and Beaudette (1982)
AQoLs	Impact of alcohol on quality of life (34 items)	0.96	Luquiens et al. (2016)
FFMQ	Total level of mindfulness	.88^a^	Heeren et al. (2011)
*Observation*	Noticing or attending to internal and external experiences such as sensations, thoughts, or emotions	.78	
*Description*	Labeling internal experiences with words	.88	
*Acting with awareness*	Focusing on one's activities in the moment as opposed to behaving mechanically	.89	
*Nonreactivity*	Allowing thoughts and feelings to come and go, without getting caught up in or carried away by them	.76	
*Nonjudgment*	Taking a nonevaluative stance toward thoughts and feelings	.89	
s‐UPPS‐P			
*Negative urgency*	Proneness to act rashly in intense negative emotional contexts	.78	Billieux et al. (2012)
*Positive urgency*	Proneness to act rashly in intense positive emotional contexts	.70	
*Lack of perseverance*	Difficulty remaining focused on difficult or boring tasks	.84	
*Lack of premeditation*	Difficulty taking into account the consequences of an action	.79	
*Sensation seeking*	Openness to new experiences and preferences for risky activities	.83	
TLFB	Provides retrospective estimates of daily drinking by relying on a calendar	NA	Sobell and Sobell (1992)

Abbreviations: AQoLs, alcohol quality of life scale; BAI, Beck Anxiety Inventory; BDI‐21, Beck Depression Inventory; CEQ‐F, Craving Experience Questionnaire—frequency; CEQ‐S, Craving Experience Questionnaire—strength; FFMQ, Five Facets Mindfulness Questionnaire; NA, not applicable; s‐UPPS‐P, urgency, premeditation, perseverance, sensation seeking, and positive urgency Impulsive Behavior Scale (short version); TLFB, timeline follow‐back.

a Validation study was not conducted in the population of interest (alcohol use disorder).

### 2.4 Statistical analyses

Descriptive analyses were performed for demographics, drinking characteristics, and psychological variables. We also explored the floor and ceiling effects and the item distribution. Given the clinical nature of the sample, we a priori decided to remove any item with a floor effect >.50, these items being considered nonrelevant from the patient's perspective. Items with a floor effect of <.50 were examined one by one. Items were kept if they were judged nonredundant in comparison with other items and considered to explore a well‐documented trigger of alcohol consumption and thus were theoretically relevant for inclusion in the final version of the TCTQ. We report here, in accordance with the consensus‐based standards for the selection of health measurement instruments checklist (Mokkink et al., 2010), internal consistency, structural and construct validity, and hypothesis testing.

### 2.5 Validity

#### 2.5.1 Structural validity

Only patients who completed all items of the final version of the TCTQ were included in the structural validity analysis. No data imputation was performed. The TCTQ is based on a reflective model, implying that a latent variable is posited as the common cause of items. Exploratory factor analysis (EFA) was conducted to determine the dimensional structure of the TCTQ. The optimal number of factors was identified from a preliminary principal component analysis, using inspection of Cattell's scree plot for the point of inflection (Cattell, 1966), the simulation method of 40 data sets, and Velicer's minimum average partial (MAP) test computed on the correlation matrix (Velicer, 1976). The MAP test was bootstrapped. A first substantial dimension on the plot would graphically support the appropriateness of calculating a total score that summed all items. An EFA with Varimax rotation was performed based on the number of factors identified from the principal component analysis. The Varimax rotation was chosen by assuming that each triggering domain could potentially be independent from the others clinically and in order to force loadings on one or the other dimension from an explorative perspective. The root mean square residual was fixed as a pre‐established indicator of the goodness of fit to the data; a value of less than 0.05 is recommended (Hu & Bentler, 1999). Items with loadings below 0.40 were removed from subsequent analyses, and the EFA was repeated. Items were attributed to the dimension for which they present the highest loadings. The structure presented below (see Table 4) is the final one retained following item selection. Item–dimension correlations were computed, omitting the item from its dimension, in order to avoid artificially inflated correlation (package psych R; Streiner & Norman, 2008). The total score was obtained by summing all remaining items after removal of items with a high floor effect and with low loadings on all factors.

#### 2.5.2 Construct validity and hypothesis testing

To assess construct validity, we conducted Spearman correlations between the TCTQ total and dimension scores and craving (CEQ scores), drinking characteristics, anxiety/depression (Beck Anxiety Inventory, Beck Depression Inventory), impulsivity (s‐UPPS‐P scores), and mindfulness traits (FFMQ scores). We expected a positive and moderate‐to‐high correlation between the TCTQ total score/subscores and drinking outcome and craving intensity and frequency. We further expected a positive moderate correlation between the TCTQ total score/subscores and impulsivity, anxiety, and depression scales. We expected a negative and moderate correlation between the TCTQ total/subscores and mindfulness, in particular a stronger correlation with the nonreactivity subscale. We also expected a positive moderate correlation with the impact of alcohol on quality of life, as assessed with the alcohol quality of life scale.

#### 2.5.3 Internal consistency

Internal consistency was assessed for each dimension of the TCTQ and for the total score by using Cronbach's coefficient alpha.

All analyses were performed with R 3.4.4 software.

## 3 RESULTS

### 3.1 Sample description

We included 111 patients with current AUD between October 2015 and July 2018. The mean age was 48.8 years and 68% were male. Forty‐nine percent of the participants had been prescribed addictolytic medications. Only French‐approved drugs for the treatment of AUD (maintaining abstinence or drinking reduction) were used (acamprosate, naltrexone, baclofen, nalmefene, and disulfiram). In addition, 69% of the participants had been prescribed other psychotropic medications (antidepressants, anxiolytics, mood stabilizers, and neuroleptics) (*n* = 108). The complete description of the sample is given in Table 2.

**Table 2:** Population characteristics

Characteristics	*N* = 111
Men, *n* (%)	76 (68%)
Age, mean (SD)	48.8 (10.6)
Active, *n* (%)	80 (72%)
Educational level	
High school diploma, *n* (%)	23 (21%)
Did not complete high school, *n* (%)	22 (20%)
More than high school diploma, *n* (%)	64 (58%)
Marital status	
Married, *n* (%)	44 (40%)
Single, *n* (%)	67 (60%)
Alcohol use	
Abstinent, *n* (%)	35 (32%)
Number of alcohol units over the last 30 days, mean (SD)	69.6 (98.7)
Number of participants with at least 1 HDD over the last 30 days, *n* (%)	52 (42%)
Number of HDDs over the last 30 days, mean (*SD*)	4.6 (7)
Self‐assessment	
Craving strength, CEQ‐S total, mean (*SD*)	47.3 (23.1)
CEQ‐S intensity	17.1 (8.1)
CEQ‐S imagery	16.5 (12.2)
CEQ‐S intrusion	13.6 (7.7)
Craving frequency, CEQ‐F total, mean (*SD*)	26 (23.7)
CEQ‐F intensity	9.4 (8.8)
CEQ‐F imagery	8.2 (9.3)
CEQ‐F Intrusion	8.4 (7.9)
BAI, mean (SD)	14.5 (12)
BDI‐21, mean (SD)	15.7 (9.3)
AQoLs, mean (SD)	22.9 (19)
FFMQ total, mean (SD)	119.5 (20.2)
Observation	26.7 (5.7)
Description	24.3 (6.8)
Acting with awareness	26.4 (6.5)
Nonreactivity	18.5 (4.7)
Nonjudgment	22.7 (6.7)
s‐UPPS‐P	
Negative urgency	10.1 (3)
Positive urgency	10.9 (2.6)
Lack of perseverance	8 (2.8)
Lack of premeditation	7.6 (2.5)
Sensation seeking	9.3 (2.8)

Abbreviations: AQoLs, alcohol quality of life scale; BAI, Beck Anxiety Inventory; BDI‐21, Beck Depression Inventory; CEQ‐F, Craving Experience Questionnaire—frequency; CEQ‐S, Craving Experience Questionnaire—strength; FFMQ, Five Facets Mindfulness Questionnaire; HDD, heavy drinking day; s‐UPPS‐P, urgency, premeditation, perseverance, sensation seeking, and positive urgency Impulsive Behavior Scale (short version).

### 3.2 Item description

Items are described in Table 3. A floor effect ≥.50 was found for 16 items. Despite the floor effect, one item (Item 2) was kept for analyses for theoretical reasons, given the important literature suggesting that exposure to visual cues can trigger craving (Carter & Tiffany, 1999; George et al., 2001; Thomas et al., 2005; Witteman et al., 2015). Moreover, no other item explored this very aspect. The other 15 items with a floor effect were removed for the analyses that followed. Most of these items, 12 of the 15, relate to sensorial or physical features: Items 4, 6, 9, 11, 13, 15, 20, 21, 31, 34, 37, and 40. The three remaining items with a high floor effect were Items 29 (“proud and self‐confidence”), 16 (“thoughts related to people”), and 32 (“talk related to alcohol”). Three more items, 5 (“overrated control over drinking”), 17 (“physiological needs”), and 26 (“physical weakness”), were removed because of low loadings in all three factors.

**Table 3:** Item description

			Response options		
Item number	Mean	SD	Ceiling effect	Floor effect	NA
Item 1 “boredom”	3.89	1.76	0.25	0.13	6
Item 2 “visual cues”	2.19	1.58	0.03	0.54	4
Item 3 “pleasure”	4.07	1.64	0.24	0.10	6
Item 4 “heart rate”	2.03	1.51	0.04	0.62	6
Item 5 “overrated control”	2.55	1.62	0.05	0.39	7
Item 6 “sounds”	1.50	1.05	0.01	0.75	5
Item 7 “stress”	4.65	1.62	0.44	0.09	7
Item 8 “relief”	3.66	1.79	0.22	0.18	5
Item 9 “headache”	1.33	0.88	0.03	0.83	7
Item 10 “feeling bad”	3.25	1.85	0.13	0.31	7
Item 11 “smell”	2.04	1.49	0.04	0.59	6
Item 12 “satisfaction”	3.34	1.86	0.16	0.28	6
Item 13 “salivation”	1.47	0.98	0.03	0.75	5
Item 14 “shame”	2.58	1.87	0.13	0.47	5
Item 15 “touch”	1.67	1.32	0.03	0.74	5
Item 16 “thoughts of people”	2.33	1.65	0.07	0.51	5
Item 17 “physiological needs”	2.42	1.70	0.08	0.46	5
Item 18 “arousal”	3.08	1.79	0.12	0.30	5
Item 19 “disappointment”	3.81	1.74	0.19	0.14	7
Item 20 “taste”	1.93	1.45	0.05	0.58	5
Item 21 “body temperature”	1.50	1.02	0.01	0.76	7
Item 22 “problems”	4.20	1.72	0.31	0.11	5
Item 23 “anxiety”	4.32	1.77	0.37	0.12	5
Item 24 “joy”	3.42	1.88	0.18	0.26	6
Item 25 “specific contexts”	4.02	1.85	0.30	0.18	5
Item 26 “physical weakness”	2.88	1.85	0.12	0.37	5
Item 27 “loneliness”	4.06	1.86	0.32	0.15	5
Item 28 “not well”	3.98	1.86	0.30	0.17	5
Item 29 “proud/self‐confidence”	2.25	1.70	0.08	0.54	5
Item 30 “thoughts/product”	2.90	1.77	0.07	0.36	5
Item 31 “sweat”	1.69	1.32	0.03	0.72	6
Item 32 “talk”	1.82	1.28	0.03	0.59	5
Item 33 “frustration/anger”	3.98	1.82	0.27	0.16	5
Item 34 “muscle tension”	1.98	1.43	0.05	0.56	6
Item 35 “relaxation”	3.42	1.86	0.19	0.25	6
Item 36 “thoughts/places”	2.40	1.65	0.06	0.47	5
Item 37 “tremor”	2.10	1.82	0.10	0.68	6
Item 38 “sadness/despair”	4.02	1.83	0.30	0.17	6
Item 39 “locations”	2.59	1.78	0.08	0.45	5
Item 40 “motor instability”	1.63	1.32	0.05	0.73	5
Item 41 “euphoria”	3.11	1.82	0.14	0.29	5
Item 42 “guilt”	3.31	1.80	0.14	0.24	6
Item 43 “unease”	4.11	1.82	0.32	0.16	5

*Note.* Excluded items are highlighted in gray.

Abbeviation: NA, not applicable.

### 3.3 Structural validity

**Figure 1:**
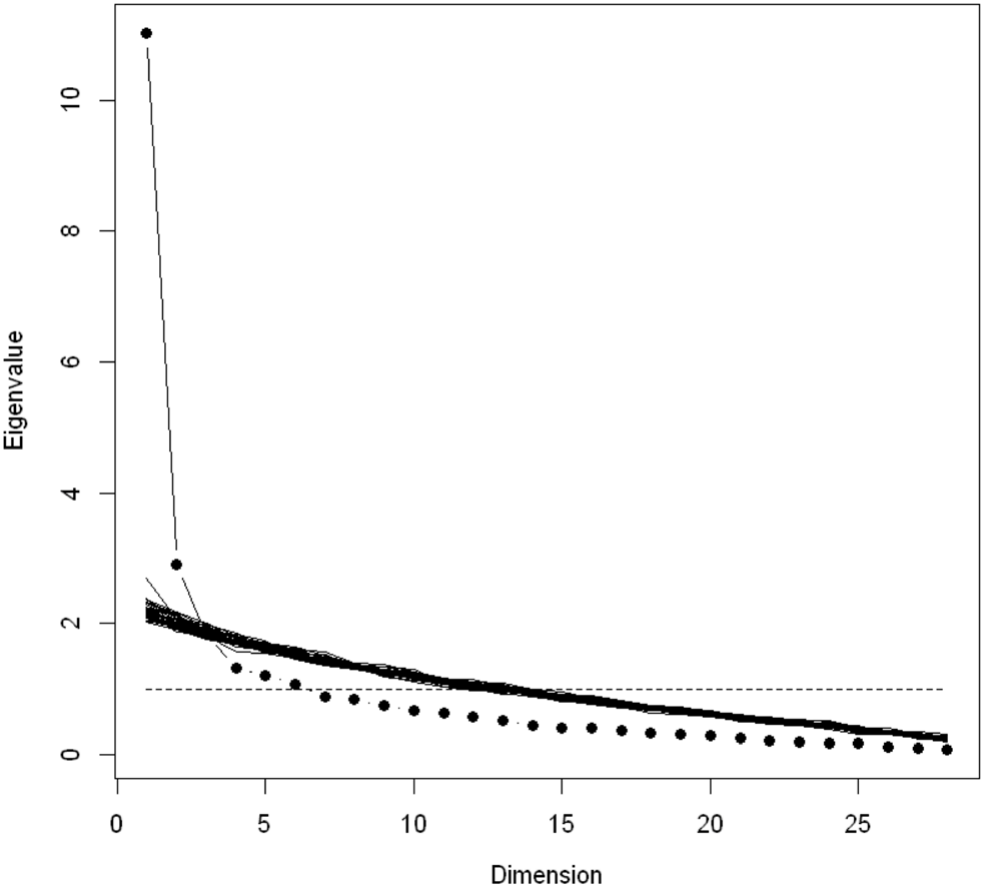
Scree plot simulation on 40 data sets

The scree plot is presented in Figure 1. The various methods used to identify the appropriate number of factors suggested a 3‐factor solution. The MAP test bootstrap results were as follows: 2: 0.11, 3: 0.39, and 4: 0.32. Low loadings were found for all factors for three items: 5 (“overrated control over drinking”), 17 (“physiological needs”), and 26 (“physical weakness”). These items were removed from the analyses that followed. Cumulative variance of the three factors resulting from the EFA on the final 25‐item questionnaire was 0.56. Loadings for the three factors are presented in Table 3.

Items 1, 7, 10, 14, 19, 22, 23, 25, 27, 28, 33, 38, 42, and 43 showed higher loadings on Factor 1. This factor includes items that explore cravings triggered by unpleasant affect. Items 3, 8, 12, 18, 24, 35, and 41 showed higher loadings on Factor 2. This factor includes items that explore cravings triggered by pleasant affect. Items 2, 30, 36, and 39 showed higher loadings on Factor 3. This factor includes items that explore cravings triggered by external alcohol cues or related thoughts (see Table 4).

**Table 4:** Item loading on factors and factor/item correlations

	Loadings on factors			Factor‐item correlation		
Item number	1 “Unpleasant affect”	2 “Pleasant affect”	3 “Cues and associated thoughts”	Factor 1	Factor 2	Factor 3
Item 1 “boredom”	**0.49**	0.04	0.42	.56		
Item 2 “visual cues”	0.14	0.13	**0.56**			.57
Item 3 “pleasure”	0.15	**0.67**	0.16		.59	
Item 7 “stress”	**0.69**	0.31	0.21	.73		
Item 8 “relief”	0.39	**0.52**	0.13		.69	
Item 10 “feeling bad”	**0.73**	0.07	0.11	.72		
Item 12 “satisfaction”	0.03	**0.78**	0.09		.76	
Item 14 “shame”	**0.40**	0.25	0.20	.50		
Item 18 “arousal”	0.32	**0.45**	0.35		.67	
Item 19 “disappointment”	**0.81**	0.18	0.06	.80		
Item 22 “problems”	**0.71**	0.25	0.18	.76		
Item 23 “anxiety”	**0.83**	0.18	0.27	.87		
Item 24 “joy”	0.22	**0.73**	0.27		.83	
Item25 “specific contexts”	**0.49**	0.31	0.20	.59		
Item 27 “loneliness”	**0.62**	‐0.06	0.40	.65		
Item 28 “not well”	**0.77**	0.14	0.19	.79		
Item 30 “thoughts/product”	0.22	0.27	**0.59**			.61
Item 33 “frustration/anger”	**0.69**	0.19	0.15	.72		
Item 35 “relaxation”	0.18	**0.64**	0.10		.72	
Item 36 “thoughts/places”	0.15	0.17	**0.76**			.63
Item 38 “sadness/despair”	**0.88**	0.13	0.11	.87		
Item 39 “locations”	0.14	0.24	**0.62**			.63
Item 41 “euphoria”	0.16	**0.60**	0.47		.78	
Item 42 “guilt”	**0.74**	0.32	0.11	.79		
Item 43 “unease”	**0.85**	0.20	0.13	.86		

*Note.* The numbers in bold represent the loading of the item in the factor it has been assigned to.

The 25‐item total score was then obtained by summing all items after removal of 15 items with a high floor effect and 3 with low loadings on all factors. The theoretical range in scores for the final 25‐item TCTQ is between 25 and 150. The mean (*SD*) total score was 87.8 (28.6). The “unpleasant affect” factor mean (*SD*) was 54.2 (19.5), with a theoretical range of 14–84. The “pleasant affect” factor mean was 24.2 (9.3), with a theoretical range of 7–42. The “cues and related thoughts” factor mean was 10.0 (5.3), with a theoretical range of 4–24.

### 3.4 Construct validity

We found no or only a very weak positive correlation between drinking characteristics and the total TCTQ score. Surprisingly, very weak positive correlations were also found between the frequency of craving and the total score and TCTQ factors. However, moderate positive correlations appeared between the strength of the last craving and the total TCTQ score. A moderate positive correlation was found between the total TCTQ score and anxiety (.42) and depression (.44) and between the impact of alcohol on quality of life and the total TCTQ score (.36). The total score for mindfulness (FFMQ) was moderately and negatively correlated with the total TCTQ score (−.35). The TCTQ total score was moderately positively correlated with urgency, premeditation, perseverance, and sensation seeking negative urgency (.40) and positive urgency (.39). Table 5 presents Spearman's correlations between the TCTQ total score/subscores and the other concepts (see Table 5).

**Table 5:** Construct validity

Assessment	TCTQ total	F1 “unpleasant affect”	F2 “pleasant affect”	F3 “external cues and associated thoughts”
HDD	.09	.07	.12	−.05
Days of use	.05	−.04	.10	−.12
Total use	.09	.02	.14	−.09
CEQ‐F	.15	.11	.08	.21
CEQ‐F intensity	.14	.11	.08	.13
CEQ‐F imagery	.12	.08	.08	.20
CEQ‐F intrusion	.19	.14	.12	.26
CEQ‐S	.42	.37	.27	.36
CEQ‐S intensity	.41	.40	.26	.20
CEQ‐S Imagery	.33	.28	.18	.36
CEQ‐S intrusion	.39	.32	.18	.32
BAI	.42	.45	.20	.25
BDI‐21	.44	.45	.13	.21
AQoLS	.36	.31	.32	.17
FFMQ total	−.35	−.35	−.17	−.18
FFMQ observation	.05	−.02	.03	.21
FFMQ description	−.13	−.10	−.06	−.14
Acting with awareness	−.31	−.28	−.22	−.23
Non‐reactivity to private events	−.17	−.22	−.00	−.07
Non‐judgment	−.48	−.46	−.30	−.31
UPPS‐P negative urgency	.40	.44	.22	.15
UPPS‐P Positive urgency	.39	.34	.27	.29
UPPS‐P Lack of perseverance	.16	.13	.04	.24
UPPS‐P Lack of premeditation	−.05	−.02	−.05	−.01
UPPS‐P Sensation seeking	.24	.25	.11	.31

Abbreviations: AQoLs, alcohol quality of life scale; BAI, Beck Anxiety Inventory; BDI‐21, Beck Depression Inventory; CEQ‐F, Craving Experience Questionnaire—frequency; CEQ‐S, Craving Experience Questionnaire—strength; FFMQ, Five Facets Mindfulness Questionnaire; HDD, heavy drinking day; s‐UPPS‐P, urgency, premeditation, perseverance, sensation seeking, and positive urgency Impulsive Behavior Scale (short version); TCTQ, Transaddiction Craving Triggers Questionnaire.

Cronbach's coefficient alpha for the TCTQ total score was 0.95, which shows excellent internal consistency. Cronbach's coefficients for Factors 1, 2, and 3 were 0.95, 0.86, and 0.80, respectively.

## 4 DISCUSSION

In the current study, we aimed to develop a new scale that measures sensitivity to craving triggers, that is, the TCTQ, and to investigate its psychometric properties in AUD. Results showed that a shortened 25‐item version of the TCTQ has good psychometric properties in AUD and presents three specific dimensions, namely, (a) unpleasant affect, (b) pleasant affect, and (c) external cues and related thoughts. The structure was highly simplified in comparison to the seven a priori hypothesized dimensions proposed in the framework during scale development, which included external cues, anticipatory responses, associated thoughts, physiological deficit, negative affect, positive affect, and a sense of associated deficit. Stronger support for the 3‐factor solution should be obtained by testing the resulting hypothesized model with a confirmatory factor analysis in a new sample in the future.

A number of items were removed from the initial 43 items, based on factor loadings and analysis of floor effects; all of these items except one (visual cues) explored sensorial or physical triggers that did not appear to be relevant for most included AUD patients. Notably, all sensorial and physical items appeared to be irrelevant as triggers in our AUD sample. Indeed, these items represented the set of items of the a priori thought dimensions: physiological deficit, anticipatory responses, and external cues. The patient perspective on these sensorial items is valuable in terms of the cognitive approach of craving developed by Kavanagh et al. (2005), who suggested that “intrusive thoughts” linked to craving involve learned associations with internal or external cues. It is likely that the impact of physical sensations in triggering craving is automatic or unconscious in nature, which possibly explains why these triggers are not considered relevant from the patient's perspective in the current study. It can be hypothesized that craving triggered by neurovegetative symptoms are poorly or even not mentalized, and then objective measures such as skin conductance, salivation, temperature, respiration, heart rate, and blood pressure could be more relevant than a self‐questionnaire (Drobes & Thomas, 1999). Another item that was removed explored the possibility that a permissive thought related to overrated control over drinking could trigger craving. It may illustrate that this thought facilitates drinking without being its initial trigger. The moderate association with craving strength suggests that the subjective experience of craving cannot be equated (or is not isomorphic) with craving triggers, further justifying the relevance of measuring these two constructs separately. Notably, triggers are targeted by most empirically based psychological interventions for treating addictive disorders (Bowen, Chawla, & Marlatt, 2010; Marlatt & Gordon, 1985) but have mostly not been assessed in clinical settings given the lack of theoretically and methodologically sound instruments. The current study thus fills an important gap in the literature by providing an initial account for the psychometric properties of such an instrument in a sample of AUD patients.

The factor structure of the TCTQ shown through exploratory analysis is relevant from a clinical perspective. First, unpleasant affects have been identified as triggering craving and are known to play a pivotal role in relapse (Ehlers et al., 2018; Suter, Strik, & Moggi, 2011; Wheeler et al., 2008). Moreover, the self‐medication model of alcohol consumption is influential in the literature (Crum et al., 2013; Khantzian, 1985). The identification of two different factors that explore emotions as craving triggers, on the one hand, an unpleasant affect and on the other, a pleasant one, can be related to the cognitive theories of addiction, holding a central role in positive and negative reinforcements (and related expectancies) in the perpetuation of the addictive process. Moreover, a robust corpus of neurobiological and neurocognitive data supports the possibility that salient cues are strong craving triggers (Carter & Tiffany, 1999; George et al., 2001; Thomas et al., 2005; Witteman et al., 2015). Accordingly, we believe that assessing craving with the TCTQ could contribute to individualizing the treatment approach (e.g., if the instrument is completed during initial evaluation and case conceptualization). Moreover, the TCTQ, in particular its Factor 3 (cues and related thoughts), could constitute an ideal way to assess the effect of interventions designed to mitigate cognitive bias (C.E. Wiers et al., 2015).

Interestingly, correlations between drinking outcomes and craving triggers were lower than expected. Drinking characteristics were low in the participants who were mostly recruited during the treatment program in the clinical setting and not at the beginning of care. Furthermore, some of them probably received relapse prevention sensitization as part of their treatment. It is worth noting that the weak correlation observed between the TCTQ and other craving measures is not unexpected, given that the retrospective measurement of craving frequency or intensity over a specific period, such as the past month, may not be accurate because the occurrence of craving appears to be context dependent (Shiffman, 2000b). There was also a moderate correlation between craving triggers and the impact of alcohol on quality of life. This result could justify continuing to work on triggers, even if the behavior seems to be temporarily contained and handled, from a relapse prevention perspective and in order to improve the patient's quality of life.

Moreover, we could show that a correlation between anxiety, depression, and craving triggers especially concerned craving triggered by unpleasant feelings and emotions. This is interesting from a clinical perspective because it supports the importance of integrated care in the context of a high rate of comorbid disorders, in particular comorbid depression in AUD (Drake, Mercer‐McFadden, Mueser, McHugo, & Bond, 1998). Negative correlation with trait mindfulness encourages the implementation of mindfulness‐based strategies in relapse prevention. In AUD, repetitive heavy drinking in response to stressors and negative emotions replaces initial consumption with a conditioned and automated drinking behavior, despite related deleterious consequences (Wiers et al., 2006). Negative affect and external cues then lead automatically to subjective craving through an involuntary attentional bias toward alcohol cues (Garland, Boettiger, & Howard, 2011). Attentional bias has been shown to be positively correlated to craving (Field, Mogg, & Bradley, 2005). As a response to the uncomfortable thoughts and feelings that accompany craving, patients with AUD often try to suppress the craving to drink (Bateson, 1971). This strategy appears to be particularly counterproductive, as efforts to suppress unpleasant thoughts and feelings tend to increase them (Wegner, Schneider, Carter, & White, 1987; Wenzlaff & Wegner, 2000). Mindfulness involves nonjudgmental and nonreactive metacognitive attention to the present moment experience, without fixation on thoughts about the past or the present (Garland, 2007). It increases awareness of the presence of urges, instead of suppressing them. Mindfulness also enhances controlled cognitive operations and disrupts the atomized trigger–craving–drinking process by reducing cognitive reactivity to triggers. Mindfulness levels have been shown to be negatively correlated to attentional bias in patients with AUD (Garland, Boettiger, Gaylord, Chanon, & Howard, 2012) and to predict regulation of attentional reactivity to alcohol cues.

Furthermore, we found a positive moderate correlation of TCTQ pleasant and unpleasant affect scores with two facets of the s‐UPPS‐P scale, positive urgency and negative urgency, respectively. Negative urgency is the tendency to act rashly in response to extreme negative emotions, whereas positive urgency is the tendency to act rashly in response to positive emotions. Negative urgency has been previously shown to increase negative emotional reactivity to mood events and alcohol craving (VanderVeen et al., 2016). This correlation confirms the clinical relevance of these two TCTQ dimensions and supports the development of personalized interventions that rely on psychological functioning for relapse prevention.

Our study comes with some limitations. The sample size is relatively small, although it is adequate according to the statistical analyses conducted (Rouquette & Falissard, 2011). Because of insufficient sample size, separate factor analyses could not be conducted for subgroups, such as abstinent or nonabstinent patients. Such analyses should be performed in studies in the future. The sample is also highly heterogeneous (e.g., in terms of severity and comorbidity), as is the rule when recruitment is conducted in an ecological setting. Because our sample consisted of individuals with different drinking goals, we decided not to categorize them according to abstinence, as this would have resulted in categorizing patients as “nonabstinent” who had achieved their goal of reducing alcohol consumption. By not categorizing patients in this way, we also ensured that our study was conducted on a clinically representative sample rather than on different, not necessarily reliable, subgroups of patients (e.g., abstinent vs. nonabstinent). Further confirmatory factor analysis should be conducted to support the 3‐dimensional structure found in our study; a prospective study could also examine sensitivity, which may change the resulting calculations.

## 5 CONCLUSION

The validation process led to a shortened 25‐item version of the TCTQ in a population of patients with AUD. We found that a 3‐factor structure—unpleasant affect, pleasant affect, and cues and related thoughts—supported previous findings on the propensity of emotions and cues to trigger craving. Documentation of construct validity supported the validity of the concept of craving triggers and its close relations to psychological functioning and quality of life, rather than to drinking characteristics. The TCTQ could allow personalization of treatment according to the patient's profile and could help assess the efficacy of therapeutic interventions from a subjective perspective. The TCTQ should be validated in corresponding populations before its use in other addictions (e.g., gambling, cigarette, internet, etc.).

### DECLARATION OF INTEREST STATEMENT

C.v.H, A.C., S.R., Y.K., and J.B. declare that there is no conflict of interest. L.R. has received sponsorship to participate in scientific research funded by FRA through a convention with the University Paris Nanterre. A.B. has received sponsorship to attend scientific meetings, speaker honoraria, and consultancy fees from Bristol‐Myers‐Squibb, Lundbeck, Merck‐Serono and Mylan and is member of the invidor board. A.L. has received sponsorship to attend scientific meetings, speaker honoraria, and consultancy fees from Lundbeck and Indivior.

All authors have read and approved the manuscript for submission to the *International Journal of Methods in Psychiatric Research*; have made a substantial contribution to the conception, design, gathering, analysis and/or interpretation of data, and a contribution to the writing and intellectual content of the article; and acknowledge that they have exercised due care in ensuring the integrity of the work.

None of the original material contained in the manuscript has been submitted for consideration nor will any of it be published elsewhere except in abstract form in connection with scientific meetings.

## Supporting information

Data S1. Supporting Information
