# Supplementary material for: Psychometric properties of the transaddiction craving triggers questionnaire in alcohol use disorder
Source: Int J Methods Psychiatr Res. 2019 Dec 29;29(1):e1815. doi: 10.1002/mpr.1815 (PMC7051841; doi:10.1002/mpr.1815)
Supplement: Supplementary file 1 — Data S1. Supporting Information [file MPR-29-e1815-s001.docx]

**Transaddiction Craving Triggers Questionnaire (TCTQ)**

Ce questionnaire a pour objectif d’évaluer ce qui, pour vous, **déclenche** un désir / une envie / un besoin de consommer de l’alcool / de fumer / de jouer à des jeux de hasard et d’argent. Veuillez tout d’abord entourer l’activité sur laquelle porte ce questionnaire :

This questionnaire aims to evaluate what, for you, **triggers** a craving / an urge to drink alcohol / to smoke / to gamble. Please, let us know which activity this questionnaire focuses on:

| Boire de l’alcool | Fumer | Jouer à des jeux d’argent | Jouer à des jeux vidéo en ligne |
| --- | --- | --- | --- |
| Drink alcohol | Smoke | Gamble | Play Online Video-Games |

Veuillez indiquer dans quelle mesure les éléments suivants peuvent **déclencher**, pour vous, un désir / une envie / un besoin de vous adonner à cette activité.

Please, indicate how much the following elements can trigger, for you, a craving / an urge to indulge in the pleasure of this activity.

|  | Pas du tout |  |  |  |  | Tout à fait |
| --- | --- | --- | --- | --- | --- | --- |
|  | Not at all |  |  |  |  | Absolutely |
| 1. De l’ennui   Boredom | 1 | 2 | 3 | 4 | 5 | 6 |
| 1. Des choses que vous voyez autour de vous (par ex. publicités, ordinateur, objets associés à l’alcool / à la cigarette / aux jeux…)   Things you see around you (e.g., advertisement, objects associated to alcohol / cigarette / gambling / videogames...) | 1 | 2 | 3 | 4 | 5 | 6 |
| 1. Du plaisir   Pleasure | 1 | 2 | 3 | 4 | 5 | 6 |
| 1. Une accélération du rythme cardiaque   Heart rate increase | 1 | 2 | 3 | 4 | 5 | 6 |
| 1. Des pensées de contrôle (« je peux m’arrêter quand je le décide »)   Thoughts of control (« I can stop whenever I decide ») | 1 | 2 | 3 | 4 | 5 | 6 |
| 1. Certains sons (par ex. bruits de verres, d’un briquet, de l’argent, d’un clavier…)   Some sounds (e.g., noises of glasses, a lighter, money, a keyboard…) | 1 | 2 | 3 | 4 | 5 | 6 |
| 1. Du stress   Stress | 1 | 2 | 3 | 4 | 5 | 6 |
|  | Pas du tout |  |  |  |  | Tout à fait |
|  | Not at all |  |  |  |  | Absolutely |
| 1. Du soulagement   Relief | 1 | 2 | 3 | 4 | 5 | 6 |
| 1. Un mal de tête   Headache | 1 | 2 | 3 | 4 | 5 | 6 |
| 1. L’impression que vous vous sentez mal   The impression you feel bad | 1 | 2 | 3 | 4 | 5 | 6 |
| 1. Une odeur particulière (par ex. odeur d’alcool / de cigarette / un parfum…)   A specific smell (e.g., smell of alcohol / cigarette / a perfume) | 1 | 2 | 3 | 4 | 5 | 6 |
| 1. De la satisfaction   Satisfaction | 1 | 2 | 3 | 4 | 5 | 6 |
| 1. Une salivation accrue   An increased salivation | 1 | 2 | 3 | 4 | 5 | 6 |
| 1. De la honte   Shame | 1 | 2 | 3 | 4 | 5 | 6 |
|  | Pas du tout |  |  |  |  | Tout à fait |
|  | Not at all |  |  |  |  | Absolutely |
| 1. La sensation de toucher certains objets (par ex. un verre, un cendrier, des cartes, une souris…)   The sensation of touching some objects (e.g., a glass, an ashtray, cards, a mouse…) | 1 | 2 | 3 | 4 | 5 | 6 |
| 1. Des pensées relatives aux personnes avec qui vous buvez / fumez / jouez   Thoughts related to people with whom you drink / smoke / gamble / play videogames | 1 | 2 | 3 | 4 | 5 | 6 |
| 1. Des besoins physiologiques (par ex. faim ou soif)   Physiological needs (e.g., hunger or thirst) | 1 | 2 | 3 | 4 | 5 | 6 |
| 1. De l’excitation   Arousal | 1 | 2 | 3 | 4 | 5 | 6 |
| 1. De la déception   Disappointment | 1 | 2 | 3 | 4 | 5 | 6 |
| 1. Un goût spécifique (par ex. une boisson, un chewing-gum, des biscuits apéritifs, de pizza…)   A specific taste (e.g., a drink, a chewing gum, salted biscuit, pizza…) | 1 | 2 | 3 | 4 | 5 | 6 |
|  | Pas du tout |  |  |  |  | Tout à fait |
|  | Not at all |  |  |  |  | Absolutely |
| 1. Une variation de la température du corps (par ex. chaleur ou fraîcheur)   A body temperature change (e.g., heat or cool) | 1 | 2 | 3 | 4 | 5 | 6 |
| 1. Des pensées par rapport à des problèmes (par ex. travail, finances, une discussion sérieuse…)   Thoughts related to problems (e.g., work, finances, a serious talk) | 1 | 2 | 3 | 4 | 5 | 6 |
| 1. De la peur ou de l’angoisse   Fear or anxiety | 1 | 2 | 3 | 4 | 5 | 6 |
| 1. De la joie   Joy | 1 | 2 | 3 | 4 | 5 | 6 |
| 1. Des contextes particuliers (par ex. conflit, attente, à une fête, suite à une invitation, un jour de paie…)   Specific contexts (e.g., conflict, wait, at a party, after an invitation, a payday) | 1 | 2 | 3 | 4 | 5 | 6 |
| 1. De la fatigue physique ou un manque d’énergie   Physical tiredness or a lack of energy | 1 | 2 | 3 | 4 | 5 | 6 |
| 1. De la solitude   Loneliness | 1 | 2 | 3 | 4 | 5 | 6 |
| 1. La conscience que vous n’êtes pas bien   The awareness that you are not well | 1 | 2 | 3 | 4 | 5 | 6 |
|  | Pas du tout |  |  |  |  | Tout à fait |
|  | Not at all |  |  |  |  | Absolutely |
| 1. De la fierté ou de la confiance   Pride or confidence | 1 | 2 | 3 | 4 | 5 | 6 |
| 1. Des pensées portant sur l’alcool, les cigarettes, les jeux   Thoughts about alcohol, cigarettes, gambling, videogames | 1 | 2 | 3 | 4 | 5 | 6 |
| 1. De la transpiration   Sweat | 1 | 2 | 3 | 4 | 5 | 6 |
| 1. Une discussion portant sur l’alcool, la cigarette ou les jeux   A talk about alcohol, cigarettes, gambling, videogames | 1 | 2 | 3 | 4 | 5 | 6 |
| 1. De la frustration ou de la colère   Frustration or anger | 1 | 2 | 3 | 4 | 5 | 6 |
|  | Pas du tout |  |  |  |  | Tout à fait |
|  | Not at all |  |  |  |  | Absolutely |
| 1. Une tension musculaire   Muscular tension | 1 | 2 | 3 | 4 | 5 | 6 |
| 1. De la détente   Relaxation | 1 | 2 | 3 | 4 | 5 | 6 |
| 1. Des pensées à propos de lieux où vous buvez / fumez / jouez   Thoughts about locations where you drink / smoke / gamble / play videogames | 1 | 2 | 3 | 4 | 5 | 6 |
| 1. Des tremblements   Shivers | 1 | 2 | 3 | 4 | 5 | 6 |
| 1. De la tristesse ou du désespoir   Sadness or despair | 1 | 2 | 3 | 4 | 5 | 6 |
| 1. Des endroits particuliers (par ex. dans un bar, un casino, un bureau …)   Specific locations (e.g., in a bar, a casino, an office…) | 1 | 2 | 3 | 4 | 5 | 6 |
| 1. Un tic nerveux ou un mouvement rapide et répété (par ex. tapotement, jambe qui tremble…)   A tic or a twitch (e.g., tapping, shaking leg) | 1 | 2 | 3 | 4 | 5 | 6 |
| 1. De l’euphorie   Euphoria | 1 | 2 | 3 | 4 | 5 | 6 |
| 1. De la culpabilité   Guilt | 1 | 2 | 3 | 4 | 5 | 6 |
| 1. Un sentiment de mal être   A feeling of unease | 1 | 2 | 3 | 4 | 5 | 6 |

Indices Externes – External Cues 🡪 2, 6, 11, 15, 20, 25, 32, 39

Réponses Anticipatrices – Anticipatory Responses 🡪 4, 13, 21, 34, 40

Pensées Associées – Associated Thoughts 🡪 5, 16, 22, 30, 36

Affects Négatifs – Negative Affect 🡪 1, 7, 14, 19, 23, 27 33, 38, 42

Déficits Physiologiques – Physiological Deficit 🡪 9, 17, 26, 31, 37

Affects Positifs – Positive Affect 🡪 3, 8, 12, 18, 24, 29, 35, 41

Sentiment de manque – Sense of Associated Deficit 🡪 10, 28, 43
